# Positron emission tomography imaging of lung cancer: An overview of alternative positron emission tomography tracers beyond F18 fluorodeoxyglucose

**DOI:** 10.3389/fmed.2022.945602

**Published:** 2022-10-05

**Authors:** Jing Zhu, Fei Pan, Huawei Cai, Lili Pan, Yalun Li, Lin Li, YunChun Li, Xiaoai Wu, Hong Fan

**Affiliations:** ^1^Department of Respiratory and Critical Care Medicine, West China Hospital, Sichuan University, Chengdu, China; ^2^Respiratory and Critical Care Medicine, Mianyang Central Hospital, School of Medicine, University of Electronic Science and Technology of China, Mianyang, China; ^3^NHC Key Laboratory of Nuclear Technology Medical Transformation, Mianyang Central Hospital, School of Medicine, University of Electronic Science and Technology of China, Mianyang, China; ^4^Department of Nuclear Medicine, Laboratory of Clinical Nuclear Medicine, West China Hospital, Sichuan University, Chengdu, China; ^5^Department of Nuclear Medicine, The Second People’s Hospital of Yibin, Yibin, China

**Keywords:** PET, lung cancer, ^18^F-FDG, radiotracer, tyrosine kinases inhibitor

## Abstract

Lung cancer has been the leading cause of cancer-related mortality in China in recent decades. Positron emission tomography-computer tomography (PET/CT) has been established in the diagnosis of lung cancer. ^18^F-FDG is the most widely used PET tracer in foci diagnosis, tumor staging, treatment planning, and prognosis assessment by monitoring abnormally exuberant glucose metabolism in tumors. However, with the increasing knowledge on tumor heterogeneity and biological characteristics in lung cancer, a variety of novel radiotracers beyond ^18^F-FDG for PET imaging have been developed. For example, PET tracers that target cellular proliferation, amino acid metabolism and transportation, tumor hypoxia, angiogenesis, pulmonary NETs and other targets, such as tyrosine kinases and cancer-associated fibroblasts, have been reported, evaluated in animal models or under clinical investigations in recent years and play increasing roles in lung cancer diagnosis. Thus, we perform a comprehensive literature review of the radiopharmaceuticals and recent progress in PET tracers for the study of lung cancer biological characteristics beyond glucose metabolism.

## Introduction

Lung cancer (LC) is a major threat to public health, accounting for the highest cancer-related mortality among all types of cancers worldwide (1, 2). Based on histological differences, LC can mainly be classified into small-cell lung carcinoma (SCLC), non-small-cell lung cancer (NSCLC) and pulmonary neuroendocrine tumors (NETs) (3, 4). NSCLC accounts for approximately 85–90% of all LC incidences and includes squamous-cell carcinoma (SCC), adenocarcinoma, and large-cell carcinoma (5). In the last century, the overall 5-year survival rates for LC patients remain relatively low, since LC is often advanced to the late stage when diagnosed and the treatments are limited under those circumstances (2, 6). Fortunately, inspiring improvements in survival rates of LC have been achieved in recent decades, which benefited from the development of molecular imaging technologies, including positron emission tomography (PET) (7, 8).

Compared with conventional X-ray and CT, PET has the advantages of tumor targeting and effective quantitative capabilities and thus is more powerful in detecting functional abnormalities in lung cancer diagnosis (9–11).

As an analog of glucose, ^18^F-fluorodeoxyglucose (^18^F-FDG) is the most commonly used PET tracer for the detection of solid tumors. ^18^F-FDG accumulates in tumor cells *via* membrane glucose transporters (GLUT-1 and GLUT-3) due to abnormally increased glucose metabolism (^18^F-FDG can be phosphorylated by hexokinase and the product is more polar that can be trapped in the tumor cell, and the cellular concentration of ^18^F-FDG can be visualized by PET and represent the level of glucose metabolism) (12). Therefore, ^18^F-FDG PET is widely used in the clinic as a revolutionary imaging technique for tumor diagnosis, staging, treatment planning and prognosis assessment. The diagnostic and prognostic value of ^18^F-FDG PET in LC patients has been extensively investigated, and studies have also shown the effectiveness of ^18^F-FDG for NSCLC staging (13–15).

However, since tissue glucose metabolism is not malignancy specific, other conditions, such as inflammatory/infective processes, will also cause increased ^18^F-FDG uptake and false-positive results (16, 17). Furthermore, a great amount of tumor heterogeneity can be found in all histologic subtypes of LC, and ^18^F-FDG uptake is variable in different subtypes: SCC displays higher FDG-avid than adenocarcinomas, while pulmonary NETs, lepidic predominant adenocarcinomas and mucinous neoplasms usually show relatively low ^18^F-FDG uptake (18, 19). Heterogeneity may present in different lesions within the same patient. In addition, multiple microenvironmental factors in different stages of LC, such as hypoxia and tumor angiogenesis, may also affect tumor progression as well as treatment response. Therefore, ^18^F-FDG PET is not able to provide full information about growth and metabolism of tumors, such as cell proliferation rate, expression of certain receptors, protein synthesis and amino acid metabolism, angiogenesis, etc., and this information are also important for diagnosis and treatment of tumors. To fully investigate tumor features for precise individual treatment, more specific PET radiotracers are required for characterizing tumor pathology and monitoring/predicting the therapeutic response (20).

In past decades, great efforts have been made to develop novel PET tracers to improve the specificity and sensitivity of PET imaging of LC. Although most of these newly reported PET tracers are in the early research stages, several tracers have entered clinical investigations, which hold the unlimited potential of clinical value for diagnostic PET imaging. This paper reviews the recent progress in PET tracers used in LC other than FDG, with their development history, preclinical and clinical results, and potential for future applications.

## Imaging of cellular proliferation

Uncontrolled cell proliferation can be found in all subtypes of lung cancer and is regarded as the key prognostic predictor of malignancy. As a ribosomal RNA transcription-related nuclear protein, Ki-67 is highly expressed during the dividing phases of the cell cycle (S, G1, G2, and M), plays a crucial role in cancer metastasis and can be used as a biomarker for the detection of various tumors. Patients with LC who possess high levels of Ki-67 expression are mostly related to poor differentiation, decreased progression-free survival (PFS), and decreased overall survival (OS) (21–23). Studies have shown that ^18^F-FDG uptake correlates strongly with Ki-67 expression (percentage of positive cells); however, ^18^F-FDG does not directly target the process of cell proliferation (23–27).

**FIGURE 1:**
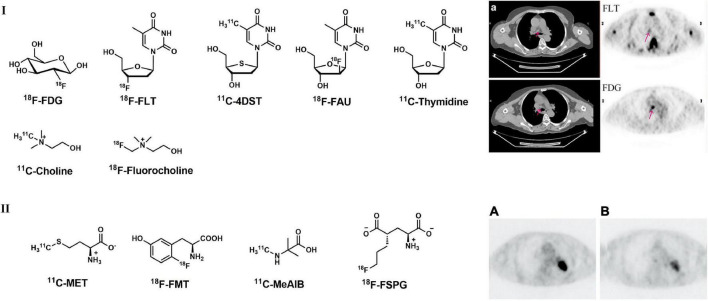
Structures of representative PET tracers for cellular proliferation, amino acid metabolism and related representative PET images in LC patient. **(I)** Typical PET and CT images of a 60-year-old patient (female) with adenocarcinoma with ^18^F-FLT **(upper)** and ^18^F-FDG **(lower)**; **(II)** ^18^F-FDG **(A)** and ^18^F-FMT **(B)** images in 74-year-old patient (male) with lung adenocarcinoma.

In recent decades, a series of ^18^F-labeled thymidine analogs, such as ^18^F-FLT, have been further incorporated into DNA *via* DNA synthesis procedures (^18^F-FAU, ^11^C-DST, etc.) (Figure 1), were successfully developed as cellular proliferation PET imaging probes. These analogs follow the same salvage pathway as thymidine, in which they were phosphorylated to thymidine monophosphates by upregulated thymidine kinase 1 and hence trapped in cells during S-phase. With these specially designed PET tracers, the cellular proliferating capability of LCs can be directly visualized.

Among these thymidine analogs, ^18^F-FLT is the most widely used tracer in the clinic. ^18^F-FLT accumulation in tumor cells correlates with histopathological Ki-67 expression and tumor angiogenesis (28, 29). A study carried out by Buck et al. indicated that 18F-FLT uptake and distribution were related exclusively to malignancies; however, 4 out of 8 benign lesions showed positive ^18^F-FDG uptake (30). Similar studies were carried out using both ^18^F-FDG and ^18^F-FLT thereafter, and the results indicated that ^18^F-FLT is more specific and more accurate (at least equally) in the detection of primary LC (31, 32). Compared with ^18^F-FDG, ^18^F-FLT is more specific and sensitive in the detection of primary tumors but shows less accuracy for N staging in LC patients (28, 33, 34). Typical PET images of ^18^F-FLT in LC patient were presented in Figure 1 (34).

Trigonis found that a significant decrease in FLT uptake was observed in NSCLC patients (stages I to III) after radiation therapy or radical chemoradiation therapy, which is more sensitive than ^18^F-FDG (35, 36). Therefore, ^18^F-FLT shows promising properties in evaluating treatment responses. Furthermore, Kobe et al. found that lower residual ^18^F-FLT and ^18^F-FDG uptake were associated with improved PFS in NSCLC patients who had received erlotinib therapy, indicating that ^18^F-FLT had prognostic value in LC patients (37, 38).

Based on the observations of 4′-methyl-[^14^C]-thiothymidine with its fast accumulation in rapidly proliferating tissues, ^11^C-labeled 4′-methyl-thiothymidine (^11^C-4DST, Figure 1) was synthesized and evaluated *in vitro* (39). In a rodent model, ^11^C-4DST showed high tumor uptake (sensitivity) and selectivity, with a tumor SUV_*max*_ of 4.93. The tumor-to-muscle ratio is 12.7, which is similar to that of ^18^F-FDG (13.2) (40). A recent study indicated that ^11^C-4DST displayed a higher correlation with proliferation of lung cancer, and the correlation coefficient between SUV_*max*_ and Ki-67 expression was significantly higher with ^11^C-4DST (0.82) than with ^18^F-FDG (0.71) in 18 NSCNC patients (41). As an unnatural analog of thymidine, FMAU was first evaluated under clinical investigations for the treatment against hepatitis B virus (HBV) (42). Then, L-FMAU was also radiolabeled with ^18^F and indicated high tumor uptake in H441 animal models; however, high physiological uptake in the liver and kidneys was investigated in human studies (42, 43).

Although many other radiolabeled thymidine analogs targeting cellular proliferation and DNA synthesis have been reported in recent years, few tracers have entered clinical evaluations. For example, ^11^C-thymidine (Figure 1) showed rapid metabolism and non-specific binding in the bone marrow and liver, indicating that this tracer is not suitable for further clinical use (44). The high concentrations of ^18^F-FAU in the circulating system and the high activity accumulation in skeletal muscle also limit its application as PET probes (45).

As a quaternary ammonium base, choline is a precursor for the synthesis of cell membrane phospholipids. After phosphorylation, choline is incorporated into phosphatidylcholine and undergoes its metabolic pathway (46). Increased choline metabolism can be observed in oncogenesis and tumor growth processes, as well as tumor proliferation (47). Radiolabeled with ^11^C or ^18^F choline analogs have been widely used in PET imaging for the detection of neoplastic tissues (48). ^11^C/^18^F-choline (Figure 1) showed good performance in many malignant tumors, including LC (48). Although choline analogs usually showed false negatives with low degree of malignancy and highly differentiated neoplasms (49, 50), in solitary lung nodules it was demonstrated that some benign conditions like granulomatous inflammation choline is negative and ^18^F-FDG is falsely positive. In addition, choline-based PET may be superior to ^18^F-FDG PET for the diagnosis of granulomatous lymph nodes and lymph node metastasis (49, 51).

Although no relevant guideline has recommended PET imaging of proliferation for diagnosis of tumors, ^18^F-FLT was the most widely used tracer in clinical investigations and applications.

## Imaging of amino acid metabolism and transportation

Beyond glucose, an abundant supply of amino acids is important for cancer cells to sustain their proliferation activities. Amino acids not only play crucial roles in nucleosides and protein synthesis for the maintenance of cellular homoeostasis but also serve as important suppliers for energy metabolism (52). In most LC malignancies, upregulated amino acid transportation and metabolism can be observed. Therefore, PET imaging of amino acid transport and metabolism showed effectiveness in diagnosing tumors.

As an important intermediate in phospholipidic biosynthesis in lung cancer cells, methionine (MET) can directly reflect amino acid transportation; therefore, ^11^C-MET (Figure 1) can be used as an effective PET tracer for detecting tumors. According to a variety of past studies, ^11^C-MET PET/CT is more specific and sensitive than ^18^F-FDG in differentiating benign lesions and lung cancer in pneumoconiosis (53–57). In addition, with the advantage of low physiological uptake in the brain, ^11^C-MET PET/CT showed better efficacy in detecting LC with brain metastasis (58, 59).

**FIGURE 2:**
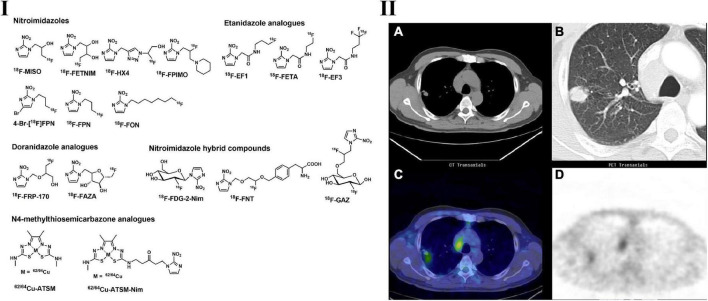
Structures of representative PET tracers for tumor hypoxia **(I)** and typical PET and CT images of ^62^Cu-ATSM in patient with lung adenocarcinoma **(II)**. **(IIA,B)** CT images indicated the mediastinal lymphadenopathy and lung tumor located in right upper lobe; **(IIC,D)** ^62^Cu-ATSM PET and PET/CT images shows high radioactivity accumulation in the enlarged lymph node and lung tumor.

Other radiolabeled amino acids, such as L-[3-^18^F]-alpha-methyltyrosine (^18^F-FMT, Figure 1) and 2-amino[^11^C]methyl-isobutyric acid (^11^C-MeAIB, Figure 1), were also developed for the detection of LC in the last decade (60). Mori et al. found that ^18^F-FMT uptake is related to proliferative activity and tumor angiogenesis in NSCLC and could serve as an independent prognostic factor for patients carrying pulmonary adenocarcinoma (60, 61). ^18^F-FMT uptake is strongly correlated with amino acid transporter (LAT1) and holds higher specificity in tumors than in peripheral organs, which makes ^18^F-FMT a promising PET tracer for detecting amino acid transportation in LC (62). In addition, ^18^F-FMT also showed prognostic value for OS in NSCLC patients according to past research (Figure 1II) (63, 64). Nishii et al. found that ^11^C-MeAIB PET achieved better capability than ^18^F-FDG in differentiating benign and malignant pulmonary and mediastinal mass lesions and better accuracy than ^11^C-MET in diagnosing brain tumors (65). As a glutamic acid derivative, ^18^F-labeled (S)-4-(3- -fluoropropyl)-l-glutamic acid (^18^F-FSPG, BAY 94-9392, Figure 1) also showed promising results in the detection of malignant diseases, including NSCLC (66).

Beyond those tracers, there have been many reports about radiolabeled amino acids targeting protein synthesis and amino transportation pathways, along with their applications in detecting tumors in recent decades, such as ^18^F-FET, ^18^F-FACBC, ^11^C-ACBC, ^11^C-ACPC ^11^C-AMT, etc. Those tracers have also been investigated in LC patients in clinic, but the details are not discussed because of the scope of this paper (67–70).

According to the joint European Association of Nuclear Medicine (EANM)/European Association of Neuro-oncology (EANO)/Working group of Response Assessment in Neuro-Oncology (RANO) practice guidelines and Society of Nuclear Medicine and Molecular Imaging (SNMMI) standard procedures, ^11^C-MET, ^18^F-FET yielded high-quality imaging standard by PET with patients with glioma as well as ^18^F-FDG (71).

## Imaging of tumor hypoxia

Oxygen is crucial for cellular energy metabolism. However, hypoxia was observed in a variety of solid tumors and was regarded as an important biological feature (72). Hypoxia is also considered to be associated with chemotherapy resistance and radiotherapy resistance. Several studies have shown that imaging assessments of hypoxia may hold great value to select patients who would benefit from individualized targeted therapy utilizing the presence of hypoxia (72). With the help of specially designed radio-labeled tracers, PET imaging of tumor hypoxia may provide non-invasive, repeatable images with high spatial resolution and high sensitivity against hypoxic regions in LC. PET hypoxia tracers can be classified into two groups according to their structures: (1) Radiolabeled nitroimidazole analogs; (2) metal chelates.

### Nitroimidazole analogs

Nitroimidazoles can be reduced into reactive intermediary metabolites in the presence of intracellular reductases, and this process is directly regulated by the level of hypoxia. Subsequently, nitroimidazole undergoes a futile reduction cycle and returns to the original structure with sufficient competitive electron acceptors. Otherwise, nitroimidazole compounds will be trapped in the hypoxic cells through the formation of hydroxylamine alkylating agents after a reduction reaction (73). Therefore, this process can be visualized by radiolabeled nitroimidazole compounds via PET imaging.

According to clinical research, tumor hypoxia is highly variable and widely prevalent. ^18^F-FMISO (Figure 2) was first synthesized in 1986 and is now the most widely used derivative of nitroimidazole (74). Kubota et al. discovered increased ^18^F-FMISO uptake in hypoxic and radioresistant tumors in rat models (75). Then, ^18^F-FMISO accumulation was found to be well associated with the pO2 measurements in head and neck cancer and renal cell carcinoma (76–79). In preclinical studies, ^18^F-FMISO PET imaging demonstrated better utility and feasibility to identify tumor hypoxic areas compared with ^18^F-FDG PET (80–82). A variety of human tumors were evaluated by ^18^F-FMISO, and the results indicated that hypoxic regions could be effectively visualized within different tumors or different regions in the same tumor. Based on a study carried out with 8 patients bearing advanced NSCLC, ^18^F-FMISO PET can be used to define the hypoxic subareas that may correspond to the region of local recurrences. Additionally, ^18^F-FMISO uptake could be estimated as a prognostic factor and was associated with the risk of locoregional failure when used in combination with a hypoxia sensitizer (83–85). These results suggest that ^18^F-FMISO uptake may be used to predict the response to treatment and OS (86). However, with a partition coefficient (octanol/water) value of 0.44, slow whole-bode clearance and low contrast of ^18^F-FMISO PET images were observed, making ^18^F-FMISO criticized and not favorable for clinical use (72, 87).

Bearing the same scaffold of nitroimidazole, ^18^F-FETNIM (Figure 2) can specifically accumulate in hypoxic cells via the same mechanism as ^18^F-FMISO. However, ^18^F-FETNIM is more hydrophilic than ^18^F-FMISO with two hydroxyl groups and is expected to have faster clearance from well-oxygenated tissues, leading to a higher tumor-to-background (T/B) ratio than 18F-MISO in rodent models (88). In a preclinical study, ^18^F-FETNIM showed a hypoxia detection capability similar to that of 18F-FMISO in mice bearing C3H mammary carcinoma. Jinming et al. carried out a head-to-head diagnostic evaluation of ^18^F-FETNIM and ^18^F-FDG PET imaging in 26 patients with NSCLC. Both ^18^F-FDG and ^18^F-FETNIM are beneficial in the clinical evaluation of solid tumors, and 18F-FETNIM provides valuable tumor hypoxia and can be used to predict OS (89). In a study containing 32 patients bearing NSCLC, the radiotherapy response was assessed by ^18^F-FETNIM PET. They found that high uptake of ^18^F-FETNIM before radiation therapy correlates with a trend toward poor OS (90). Another study carried out by Lehtiö et al. on ^18^F-FETNIM PET in head and neck cancer demonstrated similar results (91).

Another nitroimidazole analog bearing a triazole side chain, ^18^F-HX4 (Figure 2), showed high accumulation in tumors and potential in the evaluation of tumor hypoxia. ^18^F-HX4 demonstrated faster clearance by the kidney and gallbladder and thus allowed a shorter waiting time after the injection of this tracer, making it more convenient for clinical use. The tumor-to-muscle (*T*/*M*) ratio of ^18^F-HX4 is similar to that of ^18^F-FMISO in lung cancer patients. A slight increase in the *T*/*M* ratio could be observed as the imaging time was extended. ^18^F-HX4 showed higher sensitivity and specificity to hypoxia than ^18^F-MISO and displayed higher signal-to-background contrast, which makes it more suitable for clinical use (92, 93).

To optimize the suboptimal signal-to-background ratio, more hypoxic tracers were thus developed. With the introduction of the sugar moiety, ^18^F-FAZA (Figure 2) has better hydrophilicity than ^18^F-FMISO and displayed higher tumor-to-muscle ratios (94). In preclinical and clinical studies, ^18^F-FAZA showed promising accumulation in hypoxic regions and indicated efficient prognostic value (95, 96). Fourteen patients with unresectable lung cancer underwent ^18^F-FDG and ^18^F-FAZA PET/CT on consecutive days, ^18^F-FAZA showed similar tracer uptake with ^18^F-FDG, and ^18^F-FAZA uptake in lymph nodes could be used to predict the therapy response in advanced NSCLC patients (97, 98).

Multiple nitroimidazole-based PET tracers have been developed in recent years, such as ^18^F-FPIMO, ^18^FPN, ^18^FON, 4-Br^18^FPN, ^18^F-labeled Etanidazole analogs (^18^F-EF1, ^18^F-FETA, etc.), ^18^F-labeled Doranidazole analogs (^18^F-FRP-170) and nitroimidazole hybrid compounds (^18^F-FDG-2-Nim, ^18^F-FNT, and ^18^F-GAZ) (see Figure 2). However, most of them did not show potential superiority to ^18^F-MISO to be developed as hypoxia PET tracers and need further investigation (99–104).

### Metal chelates

The typical metal chelate used in hypoxia PET imaging is radio-copper-labeled diacetyl-bis(N4-methylthiosemicarbazone) (62/64Cu-ATSM) (Figure 2) (72, 105). The accumulation of Cu-ATSM is observed in a redox environment with hypoxia, and several studies suggest that the accumulation is a result of Cu(II) to Cu(I) reduction by NADH/NADPH-based mitochondrial reduction (105). However, the precise mechanism of this tracer for the location trapping in normoxic and hypoxic regions/tissues remains uncertain. ^64^Cu-ATSM was first found to have significant hypoxia selectivity in EMT6 cells, and the selectivity was further confirmed in animal models such as 9L gliosarcoma, R3327-AT and FaDu human squamous cell rat models (106–109).

Tanaka et al. performed extensive investigations and found that ^64^Cu-ATSM uptake regions consisted of tumor cells that stayed in the cell cycle and that these cells were quiescent but clonogenic, and the regions were also hypervascular; however, ^18^F-FDG uptake regions consisted of tumor proliferative tumor cells and were hypervascular (110, 111).

Based on numerous clinical studies, ^64^Cu-ATSM PET is feasible for the detection of tumor hypoxia, including LC, and may possess prognostic value in anticancer treatments (112–115). Compared with other tumor hypoxia PET tracers, ^64^Cu-ATSM displayed a higher tumor-to-background ratio and thus provided clearer delineation of tumor regions (116). According to the ^64^Cu-ATSM PET imaging in patients with locally advanced NSCLC, quantitative and optimal semiquantitative results indicated that hypoxic burden (volume of hypoxic tumor volume * SUV_*mean*_) and hypoxic tumor volume had a significant correlation with PFS (progression of free survival) (117).

Although tumor hypoxia was proved to be crucial with tumor therapies, relevant PET tracers have not been implemented in international guidelines and radiolabeled ATSM was the preferred PET tracer in detection of tumor hypoxia in clinical studies and applications.

## Imaging of angiogenesis

Angiogenesis is associated with the formation of new blood vessels and other important physiological/pathological processes, including wound repair, physical development, reproduction, response to ischemia, solid tumor growth, and metastatic tumor spread (118). Therefore, angiogenesis has gained attention as a critical imaging target in recent years for the detection of malignant tumors, including LC (119, 120). Multiple factors have been found to be related to the complex and multistep process of angiogenesis, including vascular endothelial growth factor (VEGF), hypoxia-inducible Factor 1 (HIF-1), platelet-derived growth factor (PDGF), transforming growth factor beta (TGF-β), fibroblast growth factor-2 (FGF-2), and angiopoietins (121). Although a variety of factors exist, VEGF is considered the most important and potent factor (122, 123). In addition to those angiogenic factors, integrins have also been involved in a number of physiological/pathological processes associated with angiogenesis, including cell adhesion, differentiation, proliferation, migration, and survival (124). Specifically, as a heterodimeric cell surface receptor, α_*v*_β_3_ integrin plays a crucial role in angiogenesis by allowing the interaction between the cells and extracellular matrix and promoting the migration of endothelial cells. Therefore, VEGF and α_*v*_β_3_ integrin are regarded as the most important targets in multiple molecular imaging studies, including PET (125–127). Hence, currently focused PET tracers for *in vivo* imaging of angiogenesis can be subclassified into the following groups: (1) radiolabeled VEGF pathway inhibitors; (2) radiolabeled integrin antagonists.

Based on the reversible and irreversible binding domains to VEGF tyrosine kinase receptors, monoclonal antibodies (mAbs) displayed improvements in NSCLC patients with treatment response, PFS, and OS. Radiolabeled monoclonal antibodies are effective for the non-invasive imaging of VEGF abundant tumors and prognostic evaluation of VEGF targeted treatment (128). Luo et al. recently reported that PET imaging with ^64^Cu-NOTA-RamAb provided initial evidence for overexpression of VEGFR-2 in xenografted lung tumors, suggesting potential applications in VEGFR-2-positive lung cancers (129). The VEGF ligand family has several subtypes: VEFG-A, VEFG-B, VEFG-C, VEFG-D, VEGF-E, VEGF-F, and placental growth factor (PIGF). In addition to radiolabeled RamAb, several mAbs that target tumor-associated VEGF ligands were developed as PET tracers and evaluated in preclinical studies and clinical trials. Among those mAbs, ^86^Y-CHX-A″-DTPA-bevacizumab indicated the highest uptake in VEGF-positive tumors in MSTO-211H, SKOV-3 and LS-174T xenografted mouse models (130). In a recently performed immuno-PET trial containing 7 NSCLC patients, ^89^Zr-bevacizumab successfully visualized the tumors, and the tumor uptake indicated a positive relationship with PFS and OS (131).

**FIGURE 3:**
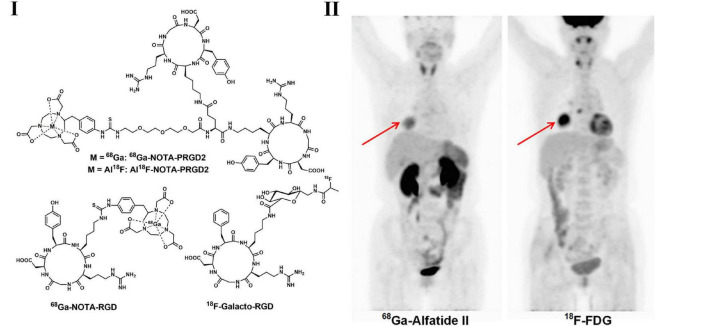
Structures of representative angiogenesis PET tracers **(I)** and PET images of ^68^Ga-Alfatide **(II)** (^68^Ga-NOTA-PRGD2) and ^18^F-FDG in patient with representative squamous carcinoma **(II)**.

Integrins are cell adhesion molecules in activated tumor endothelial cells and are considered to be involved in the differentiation, growth, migration, and neovascularization of cancer cells. With the core structure of the arginine-glycine-aspartic acid (RGD) sequence, integrin α_*v*_β_3_ binds to various extracellular matrix molecules with high specificity and affinity and plays a crucial role in the regulation of tumor growth, invasiveness and distant metastasis. Therefore, numerous integrin α_*v*_β_3_ agonists and antagonists have been developed as novel PET tracers for imaging angiogenesis (132, 133). In a clinical study with ^18^F-galacto-RGD (Figure 3) and ^18^F-FDG PET imaging of 18 patients (including 10 NSCLC patients) with primary or metastatic cancer, no significant correlation between SUVs for ^18^F-galacto-RGD and ^18^F-FDG for primary/metastatic lesions separately was observed, suggesting the complementary role of RGD-targeted PET beyond ^18^F-FDG PET (134). Zheng et al. investigated ^68^Ga-labeled RGD PET tracer in LC patients and found that ^68^Ga-NOTA-PRGD2 (Figure 3) possess higher specificity in the detection of lymph node metastasis in lung malignancies (135, 136). The recent progress of radiochemistry with ^18^F-fluoride–aluminum complexes led to the development of ^18^F-AlF-NOTA-PRGD2(^18^F-alfatide) (Figure 3) with more convenient production than ^68^Ga-labeled RGD peptides (137). In LC patients, ^18^F-alfatide PET allows specific imaging of α_*v*_β_3_ expression status with good tumor-to-background contrast and shows other promising imaging properties (137, 138). Luan et al. evaluated ^18^F-alfatide in advanced NSCLC patients before and after concurrent chemoradiotherapy (CCRT) and found that in non-responders, the SUV_*max*_ and T/NT were higher than those in responders, while the uptake ratios of tumor to normal lung could be regarded as an independent predictive factor of short-term results for CCRT in advanced NSCLC patients (139).

Although there have been plenty of PET tracers for the imaging of tumor angiogenesis reported and wildly used in clinical in recent years, none of which have been implemented in international guidelines, in spite of these tracers possess great potential in detecting EGFR status *in vivo* and personalizing therapies for NSCLC patients.

## Imaging of pulmonary neuroendocrine tumors

**FIGURE 4:**
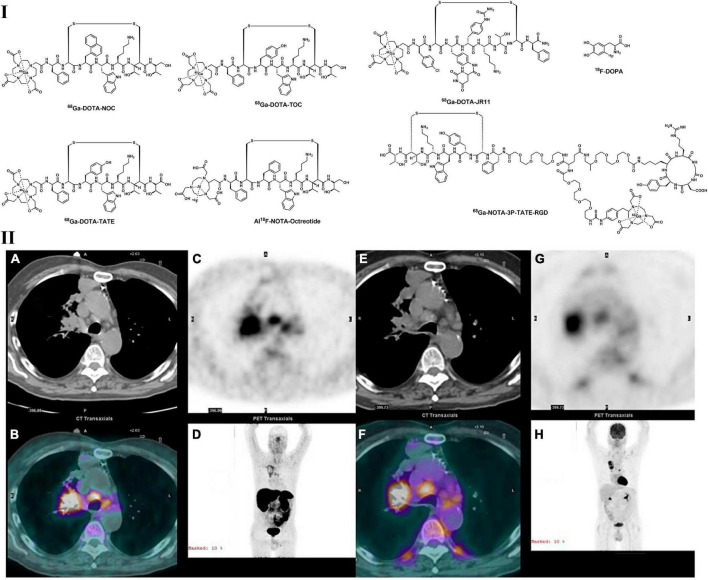
Structures of representative PET tracers for pulmonary NETs **(I)** and PET images of ^68^Ga-DOTATATE **(A–D)** and ^18^F-FDG **(E–H)** in patient with squamous cell carcinoma **(II)**. Concordant radioactivity accumulation can be observed in mediastinal adenopathy, right hilum and right upper lobe tumor.

Pulmonary NETs are a heterogeneous subgroup of malignancies that develop from a type of enterochromaffin cell named Kulchitsky cells, including low grade (typical carcinoid tumor), intermediate grade (atypical carcinoid tumor), and high-grade malignant tumors (including small cell lung cancer and large cell neuroendocrine carcinoma) (140, 141). Although high-grade pulmonary NETs tend to have FDG activity, the value of ^18^F-FDG PET for the assessment of NETs is limited (142, 143). The development of somatostatin receptor (SSTR)-based PET tracers has significantly improved the diagnosis of NETs, including LC. For example, ^68^Ga-DOTA-TATE, ^68^Ga-DOTA-TOC, and ^68^Ga-DOTA-NOC (Figure 4) showed specific binding to subtype 2 of SSTR (144). DOTA-NOC also displayed good affinities for subtypes 3 and 5 of SSTR. PET imaging with ^68^Ga–DOTA peptides offers multiple advantages compared with ^111^In–pentetreotide-based scintigraphy or SPECT, such as higher affinity to SSTRs and superior contrast and resolution, making ^68^Ga–DOTA peptides superior and convenient in the diagnosis of gastroenteropancreatic and pulmonary neuroendocrine tumors (144, 145). According to Venkitaraman et al., the specificity, sensitivity, and accuracy of ^68^Ga-DOTA-TOC PET/CT are higher than those of ^18^F-FDG PET/CT based on a prospective study with 32 patients (bronchopulmonary carcinoid suspected) (146). In a study for the detection of indeterminate pulmonary nodules, ^68^Ga-DOTATATE showed more specificity than ^18^F-FDG (147). In addition, incorporating ^68^Ga–DOTA-peptide PET imaging into ^18^F-FDG PET could enhance the specificity and sensitivity for the diagnosis of pulmonary tumors (148–150). Furthermore, indolent tumors showed low FDG uptake but high 68Ga-DOTA-TATE accumulation, indicating the diagnostic value of ^68^Ga–DOTA-peptides in the evaluation of pulmonary NETs (148).

Moreover, Zhu et al. evaluated the somatostatin receptor (SSTR) and integrin α_*v*_β_3_ dual-target PET tracer (NOTA-3P-TATE-RGD, Figure 4) in 32 patients (18 with NSCLC and 14 with SCLC). ^68^Ga-NOTA-3P-TATE-RGD showed higher uptake than ^68^Ga-NOTA-TATE in NSCLC patients, with strongly positive α_*v*_β_3_ and moderately positive SSTR2A expression detected by immunohistochemical staining. Furthermore, ^68^Ga-NOTA-3P-TATE-RGD uptake is also significantly higher than ^68^Ga-NOTA-RGD uptake in SCLC patients, with strongly positive SSTR2A and negative to mildly α_*v*_β_3_ expression (151, 152).

In addition to somatostatin analogs, radio-labeled SSTR antagonists also showed selective affinity in preclinical and clinical studies (lower affinity than that of DOTA-TATE). Compared with ^64^Cu-DOTA-TATE, ^64^Cu-NODAGA-JR11 showed much less internalization but highly strong receptor-mediated accumulation at the cell membrane (153). Specific tumor uptake of ^64^Cu-NODAGA-JR11 was also confirmed by the co-injection of unlabeled peptide (153). As indicated by a comparison study, Al^18^F-NOTA-JR11 showed superior imaging quality than ^68^Ga-DOTA-TATE in HEK293-SSTR2 tumor-bearing mice (154). Huo et al. also compared ^68^Ga-DOTA-JR11 with ^68^Ga-DOTA-TATE in patients with neuroendocrine tumors. The results indicate that although ^68^Ga-DOTA-TATE is better in the diagnosis of bone metastases, ^68^Ga-DOTA-JR11 (Figure 4) showed superior properties in the detection of liver metastases (155).

As an endogenous neurotransmitter, dihydroxy phenylalanine (DOPA) was labeled with ^18^F and used in the evaluation of the dopaminergic nervous system, as well as the detection of malignancies, including neural crest-derived (neuroendocrine) neoplasms, brain tumors and carcinoid tumors (156–159). DOPA may also accumulate in NETs because it is the substrate of dihydroxyphenol-alanine decarboxylase, which is overexpressed in NETs (160). Therefore, ^18^F-DOPA (Figure 4) PET was also used in the characterization of pulmonary nodules with neuroendocrine activities (161). However, ^18^F-DOPA showed inferior properties in detecting and staging NETs than ^68^Ga-DOTA-TATE in a comparative study performed in 25 patients (6 LC patients included) (162). This may explain why few articles could be found with ^18^F-DOPA PET used in pulmonary NETs.

Among all NET PET tracers, DOTA-TOC/NOC/TATE are the preferred PET Tracer in NET imaging as suggested by European Society for Medical Oncology (ESMO) guideline and have been widely used in clinical for the diagnosis of NETs. ^18^F-DOPA was wildly used in the diagnosis of Parkinson’s disease, and was also recommend for the imaging of glioma as a radiolabeled amino acid by EANM/EANO/RANO guidelines and SNMMI standard procedures (71).

## Imaging of tyrosine kinases in lung cancer patients

Receptor tyrosine kinases (RTKs) are transmembrane receptors in signaling pathways and play crucial roles in the tumorigenesis and pathogenesis of malignant lung cancer. RTKs are key regulators of cancer cell proliferation, differentiation, invasion, metastasis, and angiogenesis. Therefore, RTKs are also regarded as one of the most important targets for tumor treatment (163, 164). With developments in the molecular genotyping of LC (especially NSCLC), more than 50 tumor-associated RTKs have been identified, such as EGFR, c-MET, ROS1, RET, and ALK. A variety of RTK-targeting mAbs and small molecules of tyrosine kinase inhibitors (TKIs) have been developed and demonstrated inspiring clinical outcomes in patients with mutated RTKs. However, an effective patient screening and therapy prediction method is strongly required for individually targeted therapy. With radiolabeled TKIs and mAbs, visualization and quantification of tumor-specific targets become possible with PET imaging. At present, typical radiotracers that target RTKs used in LC can be subclassified into the following categories: (1) EGFR-targeted mAbs and inhibitors; (2) C-MET-targeted inhibitors; (3) VEGF-targeted mAbs and inhibitors. As VEGF is mostly associated with angiogenesis, VEGF-targeted PET tracers are discussed above.

Based on epidemiological findings, NSCLC comprises approximately 80% of all LCs and approximately 60% of NSCLC patients carrying activated EGFR (1, 3, 165). Therefore, EGFR was supposed to be one of the most important targets for *in vivo* imaging of LC. As IgG1 antibodies directed against EGFR, panitumumab and cetuximab were labeled with ^64^Cu, ^86^Y, and ^89^Zr to evaluate the imaging capability in rodent models. According to these preclinical studies, radiolabeled mAbs were found to significantly accumulate in EGFR-expressing tumors with a positive correlation with EGFR levels (166–169). ^89^Zr-cetuximab was first evaluated in 9 patients (3 head and neck cancer patients and 6 NSCLC patients), and heterogeneous uptake was observed in tumors; therefore, the predictive value of this tracer was not discussed (170). Another study containing 10 colorectal cancer patients was carried out soon. Those patients received co-injection of cold cetuximab and ^89^Zr-cetuximab and were investigated with 6 serial PET scans; 6 patients displayed increased ^89^Zr-cetuximab uptake, and 4 of these patients experienced better outcomes after cetuximab treatment. These results indicate that ^89^Zr-cetuximab PET may not only be used in the detection of EGFR *in vivo* but also be used to predict the response to cetuximab therapy (171).

**FIGURE 5:**
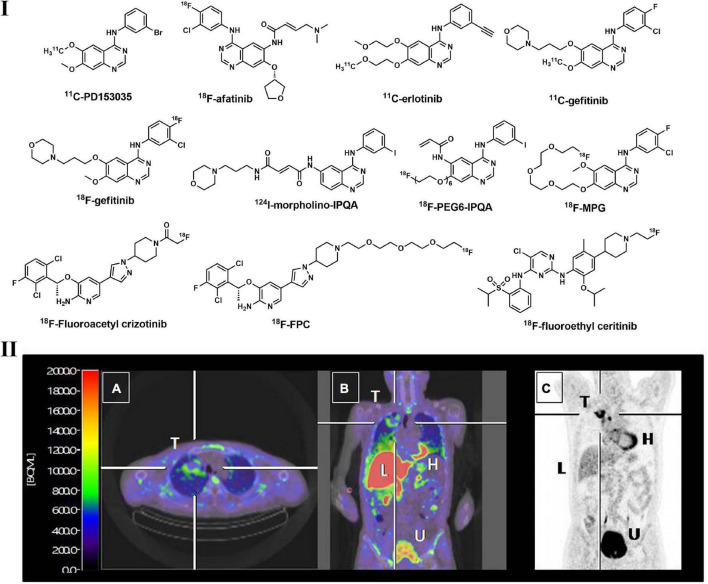
Structures of representative radiolabeled TKIs used in lung cancer imaging **(I)** and PET images of ^18^F-afatinib in a NSCLC patient with EGFR^19_*del*^ **(II)**. Tumor with EGFR^19_*del*^ can be clearly visualized by ^18^F_afatinib (T: tumor; H: heart; L: liver; U: urinary bladder).

During the last decade, radiolabeled small molecule EGFR-TKI probes have been extensively investigated in preclinical and clinical studies. As a reversible EGFR TKI, PD153035 and its analogs were labeled with ^11^C and ^18^F and evaluated *in vitro* and *in vivo*. ^11^C- PD153035 (Figure 5) showed increased uptake in EGFR-sensitive tumors in rodent models (172, 173). In a subsequent clinical study with 21 patients with advanced chemotherapy-refractory NSCLC, Meng et al. evaluated the potential of ^11^C-PD153035 for selecting patients who were likely to respond to erlotinib treatment (174). Enhanced ^11^C-PD153035 uptake in tumors prior to erlotinib treatment correlated positively with longer PFS and better OS. However, the less well-correlated survival indicated that it was not an ideal prognostic method for EGFR-TKI targeting treatment. As a successful first-generation EGFR-TKI, erlotinib was labeled with ^11^C (Figure 5) and evaluated in a variety of preclinical studies (175). All preclinical studies displayed noticeable uptake in EGFR-sensitive tumors in mice with NSCLC xenograft models, and this uptake can be effectively blocked by the cold erlotinib, suggesting the specifically saturable binding of this tracer. Memon et al. performed the first clinical study using ^11^C-erlotinib in 13 NSCLC patients, and compared with ^18^F-FDG, increased uptake of ^11^C-erlotinib can be observed in malignant lymph nodes and tumors (176).

They also found that ^11^C-erlotinib showed good blood–brain-barrier penetrability and hence is beneficial for NSCLC patients with brain metastases (177). According to a tracer pharmacokinetic analysis using the distribution volume (V_*T*_) as an uptake parameter, a 2-tissue reversible compartment model best fit ^11^C-erlotinib (178). Compared with wild-type tumors, the VT of ^11^C-erlotinib is higher in patients with EGFR exon 19 deletion, indicating that it is sensitive to EGFR mutation (179). Afatinib, a second-generation TKI that irreversibly binds to EGFR, was also labeled with ^18^F and investigated. Slobbe et al. found that ^18^F-afatinib (Figure 5) was sensitive to tumors with activated EGFR mutations in xenografted mouse models (180). ^18^F-afatinib showed higher tumor-to-background ratios in EGFR^19*del*^ and EGFR*^WT^* tumors, as well as higher stability in plasma, making this tracer more promising in clinical applications (181). As many radiolabeled EGFR TKIs with a 4-anilinoquinazoline scaffold displayed potent *in vitro* activities but showed inferior imaging properties, such as high non-specific binding, low *in vivo* stability and rapid dissociation rates, structural modifications to this scaffold were thus performed to overcome these issues, and several PET tracers were developed, such as ^18^F-PEG6-IPQA, ^124^I-morpholino-IPQA and ^18^F-MPG (Figure 5) (182–184). These tracers also displayed better uptake in tumors with EGFR mutation and lower background noise in both preclinical and clinical investigations.

In addition to the TKI PET tracers described above, several novel TKIs were radiolabeled and evaluated. However, most of them displayed inferior imaging qualities and/or were not capable of distinguishing TKI-sensitive and TKI-resistant tumors, such as ^18^F-gefitinib, which may be due to their high lipophilicity and limited tumor uptake resulting from other mechanisms (185). Overall, TKI-based PET is an important diagnostic tool for EGFR-positive lung tumors and effective clinical assessment to select those patients who would benefit more from EGFR-TKI-targeted treatment.

The ALK and HGF/c-MET pathways play significant roles in the occurrence and progression of NSCLC, indicating that these targets can be used for diagnosis and therapeutic purposes (186, 187). Accounting for approximately 5–22% of LC, c-MET-positive NSCLC patients are an important subgroup that is resistant to first- or second-generation EGFR TKIs (188). In addition, ALK-rearranged NSCLC patients comprise nearly 5–6% of all NSCLC cases (189). Studies revealed that the survival time of patients with activated c-MET mutation is shorter, suggesting that c-MET positive mutation is an adverse prognostic factor (190). Thus, c-MET-TKI/ALK-based PET imaging assessment has rapidly developed in recent years. As a potent and promising c-MET/ALK dual inhibitor, crizotinib and its analog were labeled with ^18^F for *in vivo* imaging of c-MET/ALK status (191, 192). According to Manning et al., ^18^F-fluoroacetyl crizotinib (Figure 5) showed selective binding to ALK kinase (H3122 lung cancer cells) *in vitro* (191). A polyethylene glycol (PEG)–modified crizotinib derivative (^18^F-FPC, Figure 5) was synthesized by Cheng et al. and evaluated in c-MET-positive (H1399 cell) and negative (A549 cell) NSCLC rodent models (192). Significant ^18^F-FPC accumulation in H1399 tumors was observed, indicating its potential to distinguish c-MET-positive tumors in NSCLC patients. Perera et al. synthesized ^18^F-fluoroethyl-ceritinib (Figure 5) for the evaluation of ALK expression in solid malignancies, but no *in vitro* or *in vivo* results were reported (193).

Great attention has been paid to RTK-based PET tracers as the development of RTK therapies in the last decades and over 20 PET tracers were reported and evaluated in NSCLC patients, but even the most promising tracers were still under clinical investigations and none of them have been widely accepted in clinical practices.

## Imaging of cancer-associated fibroblasts

**FIGURE 6:**
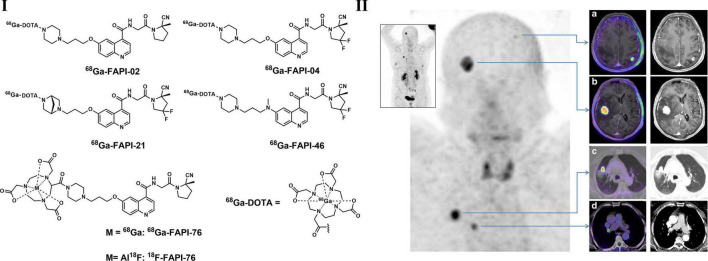
Structures of typical radiolabeled FAP inhibitors **(I)** and PET/CT images of ^68^Ga-FAPI-04 **(a–d)** in a lung cancer patient with brain metastasis **(II)**.

Cancer-associated fibroblasts (CAFs) have been proved to play important roles in several different properties of cancerous tumors, such as metastasis, migration, immunosuppression, and resistance to chemotherapy (194). Therefore, targeting CAFs may be a useful method for both diagnosis and treatment purposes. As a type II transmembrane protein expressed in activated fibroblasts, fibroblast activation protein (FAP) is highly overexpressed in a variety of malignant tumors and is related to poor prognosis, indicating that FAP is a potential target for PET imaging (195, 196). FAP imaging has been carried out with antibodies and small molecular inhibitors during the last decade (194). Several initial FAP-targeted tracers were not focused on tumor imaging; for example, radioiodine-labeled MIP-1232 was used in the detection of atherosclerotic plaques, and ^111^ In-, ^89^ Zr-, or ^99*m*^Tc-labeled antibody 28H1 was used for the imaging of rheumatoid arthritis. However, based on the structural modification of a quinoline-based FAP inhibitor and DOTA as chelator, a series of small molecule FAP PET tracers (^68^Ga-FAPIs, Figure 6) were developed and evaluated in recent years. With relatively lower lipophilicity, ^68^Ga-FAPIs showed fast body clearance and high uptake in malignant tissues according to biodistribution studies, resulting in promising high tumor-to-background images. Based on a PET imaging study in NSCLC patients, accumulated radio-labeled FAPI signals were observed not only in tumor lesions but also in active tissue remodeling sites, such as arthritis, chronic inflammation and physiological uptake in the uterus (194, 197). ^68^Ga-FAPI-04 and ^68^Ga-FAPI-46 showed the best activity against FAP and superior pharmacokinetic profiles among all reported tracers (198). According to Giesel et al., extremely high ^68^Ga-FAPI-04 uptake was found in 28 kinds of tumors, including NSCLC. FAPI was also radiolabeled with ^18^F via NOTA as a chelator to prepare ^18^F-FAPI-74. It also showed ideal imaging quality and even a lower radiation burden than ^68^Ga-FAPI-74, making FAPI-74 more flexible for clinical applications in LC (199, 200). Overall, coupling highly selective FAPIs to DOTA or other chelators, such as NOTA, FAP-targeted imaging with ^68^Ga or treatment with other therapeutic isotopes can be achieved (201).

## Imaging of other targets

In addition to the changes in the tumor microenvironment and special physiological/pathological processes described above, several other activated systems, signaling pathways and key proteins that led to the development of LC were also regarded as important targets for the discovery of novel PET tracers. These tracers have been reported for the imaging of LC and provide valuable information on target abundance and hence can be used in the determination of personalized treatment plans, as well as the prediction of therapeutic response. Several successfully developed PET tracers based on these targets used in LC patients have been reported in recent years and are briefly described in this section.

### Imaging of programmed cell death pathways

As a revolutionary cancer therapy that produces durable responses, immuno-oncology-based immune checkpoint therapy benefits patients with a variety of malignant tumors, including LC (202). Approximately 20% of NSCLC patients have achieved tumor responses when treated with immune checkpoint inhibitors. The expression of PD-1 in tumor-infiltrating lymphocytes and PD-L1/CTLA4 in the tumor cell membrane may be predictive for the response to immune checkpoint therapies. Natarajan et al. synthesized ^64^Cu-DOTA-(anti-mouse)-PD1 and evaluated it in mice xenografted with melanoma tumor cells (203). High tracer uptake was observed in tumors and lymphoid organs. The specific binding of the tracer was confirmed by bioluminescence imaging and self-blocking experiments. Ring et al. synthesized a radio-labeled PD-1 antibody named ^64^Cu–DOTA–HAC and evaluated it in mouse xenograft tumor models. ^64^Cu–DOTA–HAC showed higher tracer up in hPDL1-positive tumors compared with hPDL1-negative tumors, providing an alternative method to invasive histological and biopsy analysis to distinguish between PD-L1–positive from PD-L1–negative tumors *in vivo* (204). Yang et al. prepared ^64^Cu-NOTA-αCD276/Fab, and this probe was used in the evaluation of a CD276-targeted photodynamic therapy in NSCLC mouse models, as well as other imaging modalities for the detection of its efficacy in enhancing anti-PD-1/PD-L1 cancer therapies (205). Based on a single-domain antibody, Lin et al. synthesized a ^68^Ga-labeled PET tracer through a NOTA chelator named ^68^Ga-NOTA-Nb109 (206). According to biodistribution, autoradiography, PET imaging and immunohistochemical staining studies, ^68^Ga-NOTA-Nb109 showed specific accumulation in A375-hPD-L1 tumors with an uptake ratio of 5.0 5.0% ± 0.35% at 1 h postinjection.

**FIGURE 7:**
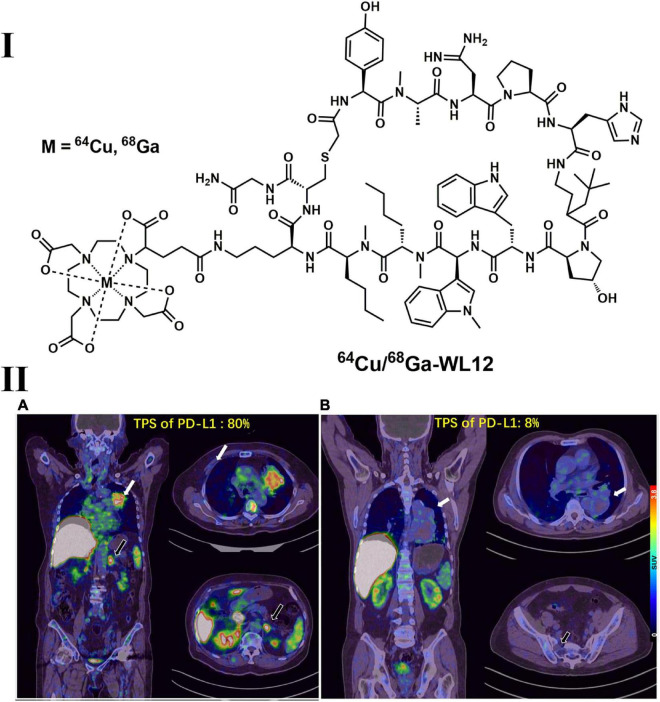
Structure of ^64^Cu/^68^Ga-WL12 **(I)** and representative PET images of ^68^Ga-WL12 in patients with NSCLC **(II)**. **(IIA)** An 80-year-old patient (female) with advanced NSCLC and a PD-L1 TPS value of 80%; **(IIB)** a 68-year-old patient (male) with a PD-L1 TPS value of 8%.

In addition, a series of peptide-based imaging agents, such as ^68^Ga-WL12, ^64^Cu-WL12 (Figure 7) and ^18^F-FPy-WL12, with high affinity (IC_50_ = 23 nM for WL12 and 26–32 nM for FPy-WL12, respectively) were synthesized and evaluated in mice bearing cancer xenografts. The results indicated that both ^68^Ga-WL12 and ^18^F-FPy-WL12 showed high tumor uptake in PD-L1-positive tumors (including NSCLC), and the uptake could be blocked by the injection of cold reference standards (207–209). Furthermore, ^68^Ga-NOTA-WL12 was recently evaluated in 9 NSCLC patients (210). After the baseline scan of ^68^Ga-NOTA-WL12 and ^18^F-FDG dual PET imaging, patients also received a combination of chemotherapy and pembrolizumab, and follow-up dual PET imaging was also performed. High contrast tumor images were obtained in ^68^Ga-NOTA-WL12 PET with tumor-to-lung ratios of 4.45 ± 1.89 at 1 h, and a strong correlation between PD-L1 expression and tracer uptake was observed, indicating potential benefits of this tracer used in clinical PD-L1 therapy (210). Although not all PD-1- and PD-L1-targeted PET tracers have been evaluated in NSCLC tumor models or patients, the significant progress in clinical outcomes achieved by anti-PD-1/PD-L1 treatments in advanced NSCLC patients will undoubtedly promote the applications of these tracers in NSCLC patients.

### Imaging of prostate-specific membrane antigen

**FIGURE 8:**
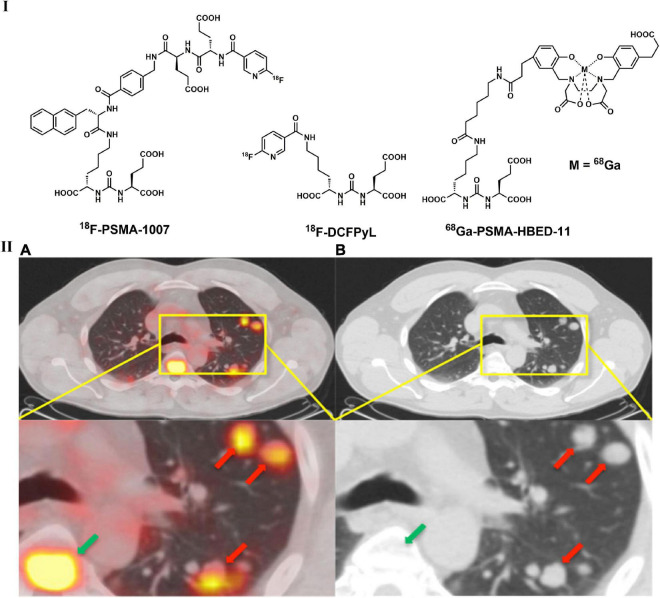
Structures of representative PSMA-targeted PET tracers **(I)** and PET/CT **(A)** and unenhanced CT **(B)** images of ^68^Ga-PSMA-HBED-11 in patient with with a recurrent prostate acinar adenocarcinoma and lung metastases **(II)**.

As a type II transmembrane protein, prostate-specific membrane antigen (PSMA) possesses glutamate carboxypeptidase/folate hydrolase activity and is a promising target for prostate cancer imaging (211, 212). A variety of radiolabeled PSMA ligands, such as ^68^Ga-PSMA-11 (Figure 8), were introduced for PET imaging, and β radionuclide ^177^Lu conjugated drugs were developed for therapy thereafter (213, 214). PSMA-targeted PET imaging and radiopharmaceutical therapy have enabled significant prostate-specific antigen imaging and therapeutic responses (215). Notably, increased ^68^Ga-PSMA uptake was not only observed in prostate cancer lesions but also found in several other benign and malignant lesions (216). According to Schmidt et al., approximately 6% of NSCLC cells express PSMA, which was mainly discovered in squamous cell carcinoma (217). In addition, the one who was diagnosed with confirmed prostate cancer had intense uptake of ^68^Ga-PSMA in lung nodules (218). Although it is not possible to easily distinguish prostate cancer lung metastases from primary lung cancers, PSMA-based PET imaging still supplies a method to seek primary tumors in the lung (217, 219, 220). By incorporating the Lys-urea-Glu motif, a variety of radiolabeled PSMA ligands (Figure 8) have been discovered in recent years, including ^11^C-MCG, ^18^F-DCFBC, ^18^F-DCFPyL, and ^18^F-PSMA-1007 (214, 215). Several studies have suggested that radiolabeled PSMA ligands can be used in the detection of prostate cancer with lung metastases, but further investigations are needed (219, 221). It is interesting that intense uptake of ^68^Ga-PSMA-11 in lung nodules was found in a male patient (diagnosed with prostate cancer), but no significant uptake of ^18^F-FDG was observed (218). Although it is not possible to easily distinguish prostate cancer lung metastases from inflammatory conditions and primary lung cancers, PSMA overexpression in LC could expand the diagnostic applications of PSMA-based PET in the clinic (217, 219, 220). In addition, ^68^Ga-PSMA-11 have been implemented in the NCCN (222).

## Conclusion

As a unique imaging modality and clinical assessment, PET/CT allows the *in vivo* detection and quantitative analysis of the desired target, as well as physiological/pathological processes at the molecular level. Although ^18^F-FDG showed high sensitivity and has been widely used in the detection, staging/restaging, treatment planning and prognosis evaluation in LC patients, a variety of other types of PET tracers were developed to investigate different aspects of the cancer microenvironment and biology and to improve tumor characterization, patient stratification, treatment response assessment and therapeutic response monitoring. These new tracers are used for the imaging of cellular proliferation, amino acid metabolism and transportation, tumor hypoxia, pulmonary NETs and special targets in LC. Although most tracers have shown promising qualities in preclinical studies, their clinical applications are limited. As the targets described above are not lung cancer specific, these tracers are also not specific to lung lesions, and not all tracers described above have been used for the detection of lung lesions. As a large number of tracers and related research articles have been reported in recent decades, not all tracers and valuable articles were included due to the scope of this paper.

With the development of targeted PET imaging and targeted therapies, more PET tracers that target specific targets, signaling pathways and tumor biology will definitely play increasingly important roles in routine clinical practice.

## Author contributions

HF, XW, and LL designed this study. YLL, HC, and YCL collected the data. JZ, FP, and LP drafted the manuscript. All authors revised this manuscript and approved for submission.
